# Emerging role of miRNAs in the regulation of ferroptosis

**DOI:** 10.3389/fmolb.2023.1115996

**Published:** 2023-02-15

**Authors:** Reza Mahmoudi-Lamouki, Sepideh Kadkhoda, Bashdar Mahmud Hussen, Soudeh Ghafouri-Fard

**Affiliations:** ^1^ Shahid Beheshti University of Medical Sciences, Tehran, Iran; ^2^ Department of Medical Genetics, Tehran University of Medical Sciences, Tehran, Iran; ^3^ Department of Pharmacognosy, College of Pharmacy, Hawler Medical University, Erbil, Iraq; ^4^ Department of Medical Genetics, Shahid Beheshti University of Medical Sciences, Tehran, Iran

**Keywords:** miRNA, ferroptosis, expression, pathwa, cancer

## Abstract

Ferroptosis is a kind of cell death which has distinctive features differentiating it from autophagy, necrosis and apoptosis. This iron-dependent form of cell death is described by an increase in lipid reactive oxygen species, shrinkage of mitochondria and decrease in mitochondrial cristae. Ferroptosis is involved in the initiation and progression of many diseases and is regarded as a hotspot of investigations on treatment of disorders. Recent studies have shown that microRNAs partake in the regulation of ferroptosis. The impact of microRNAs on this process has been verified in different cancers as well as intervertebral disc degeneration, acute myocardial infarction, vascular disease, intracerebral hemorrhage, preeclampsia, hemorrhagic stroke, atrial fibrillation, pulmonary fibrosis and atherosclerosis. miR-675, miR-93, miR-27a, miR-34a and miR-141 have been shown to affect iron metabolism, antioxidant metabolism and lipid metabolism, thus influencing all pivotal mechanisms in the ferroptosis process. In the current review, we summarize the role of microRNAs in ferroptosis and their involvement in the pathetiology of malignant and non-malignant disorders.

## Introduction

As a newly recognized kind of cell type, ferroptosis is associated with accumulation of large amounts of iron accumulation and lipid peroxidation during the process of cell death (Li J. et al., 2020). This concept has been firstly proposed by Dixon et al. as an iron-dependent way of cell death described by an increase in lipid reactive oxygen species (ROS) (Dixon et al., 2012). It has several distinctive features that distinguishes this mode of cell death from autophagy, necrosis and apoptosis (Dixon et al., 2012; Xie et al., 2016). Lack of swelling of the cytoplasm and cell organelles and absence of cell membrane splitting differentiate ferroptosis from necrosis. Moreover, absence of cell shrinkage and chromatin condensation, lack of establishment of apoptotic bodies and absence of cytoskeleton breakdown differentiate ferrptosis from apoptosis. Finally, the typical closed bilayer membrane organizations which are produced during autophagy are never seen in ferroptosis (Li J. et al., 2020). From a morphological point of view, ferroptosis is characterized by shrinkage of mitochondria and decrease in mitochondrial cristae, features which are not seen in other types of cell death (Yagoda et al., 2007; Yang and Stockwell 2008; Dixon et al., 2012). Ballooning is a specific phenotype acquired by cells during ferroptosis, defined by the establishment of a clear, rounded cell chiefly consisting of empty cytosol (Battaglia et al., 2020). Notably, during the process of ferroptosis the cell membrane is not affected, the size of nucleus is not changed, and chromatin is not condensed. However, the intracellular content of glutathione (GSH) is depleted and activity of glutathione peroxidase 4 (GPX4) is decreased. Thus, the metabolism of lipid peroxides by GPX4 is impaired. Subsequently, oxidation lipids of by Fe^2+^ in a Fenton-like mode results in production of massive quantities of ROS, which induces ferroptosis (Yang and Stockwell 2008; Angeli et al., 2014). Ferroptosis is regulated by several genetic factors, most of them being involved in modulation of iron homeostasis and lipid peroxidation (Li J. et al., 2020).

Ferroptosis happens through two main routes, i.e., the extrinsic and the intrinsic pathways. While the former is called the transporter-dependent pathway, the latter is regulated by enzymes. This process is initiated by a redox imbalance between synthesis of oxidants and antioxidants due to the abnormalities in the expressions and activities of several redox-active enzymes that synthetize or detoxicate free radicals and products of oxidation of lipids. Therefore, ferroptosis is finely regulated at several phases. This ROS-associated kind of cell death is related with two major biochemical features, i.e., iron buildup and lipid peroxidation (Tang et al., 2021).

Ferroptosis is involved in the initiation and progression of many diseases and is regarded as a hotspot of investigations on treatment of disorders (Li J. et al., 2020). Recent studies have shown that microRNAs (miRNAs) partake in the regulation of ferroptosis. The impact of miRNAs on this process has been verified in different cancers as well as non-malignant conditions. In the current review, we summarize the role of miRNAs in ferroptosis and their involvement in the pathetiology of malignant and non-malignant disorders.

### miRNAs effect on ferroptosis in cancers

miRNAs are a group of small-sized non-coding transcripts that can specifically bind with their target transcripts and induce its degradation or inhibit its translation. Through regulating several biological processes, these transcripts have fundamental roles in the process of development and cellular homeostasis (Liu et al., 2014). They can also affect expression of genes which has role in iron metabolism (Zolea et al., 2017). An experiment in melanoma cell lines has shown the role of miR-9 regulation of ferroptosis through influencing expression of GOT1. This miRNA could suppress expression of GOT1 through binding to 3′-UTR of GOT1 transcript. This binding leads to reduction of erastin- and RSL3-associated ferroptosis. On the other hand, miR-9 silencing could increase the response of neoplastic cells to erastin and RSL3. Moreover, the impact of miR-9 suppression in accumulation of lipid ROS and induction of ferroptosis can be abolished by suppression of glutaminolysis process (Zhang et al., 2018).

The Kaposi’s sarcoma herpes virus (KSHV)-encoded miRNAs have been shown to enhance expression of xCT which encodes a membrane-associated amino acid transporter. This process is mainly accomplished *via* inhibition of BACH-1, a modulator of transcription which recognizes antioxidant response elements within promoter regions. Enhancement of xCT expression by KSHV miRNAs has an important role in promotion of cell permissiveness for KSHV infection and protection of infected cells from reactive nitrogen species-induced cell death (Qin et al., 2010).

miR-17* is another miRNA that participate in the pathogenesis of cancers through influencing ferroptosis. This miRNA can inhibit activity of a number of enzymes participating in mitochondrial antioxidant pathways, namely manganese superoxide dismutase (MnSOD), GPX2 and thioredoxin reductase-2 (TrxR2). Forced up-regulation of miR-17* in PC-3 cells has decreased expressions of these antioxidant proteins through binding to their 3′-UTR. Cumulatively, miR-17* can inhibit prostate carcinogenesis *via* suppression of mitochondrial antioxidant enzymes (Xu et al., 2010a). Another experiment in prostate cancer has shown downregulation of mitochondrial antioxidant enzymes by miR-17-3p and subsequent enhancement of sensitivity of these cells to radiation (Xu et al., 2018).

Another study in multiple myeloma has shown that miR-17-5p regulates expression of the iron exporter ferroportin (FPN1), promote cell proliferation, enhance cell cycle progression, and suppress apoptosis. Expression of miR-17-5p is suppressed by the transcription factor Nrf2. Nrf2 also decreases FPN1 expression and enhanced accumulation of iron and production of ROS in the cells (Kong et al., 2019).

miR-18a is another miRNA which is involved in the regulation of ferroptosis. This miRNA has been shown to suppress expression of ALOXE3 in glioblastoma cells. Besides, ALOXE3 knock-down has enhanced secretion of 12-HETE from glioblastoma cells, decreasing migration of these cells through activation of GsPCR/PI3K/Akt axis (Yang X. et al., 2021).

miR-20a has also been shown to regulate expression of FPN through binding to its 3′-UTR. Experiments in lung cancer cells have shown that down-regulation of FPN increases cell proliferation and colony formation, most probably through enhancing iron accessibility for neoplastic cells (Babu and Muckenthaler 2016).

In liver cancer cells, miR-18a has been shown to reduce expression of GCLC- a gene that regulates biosynthesis of glutathione. miR-18a also reduces GSH levels in tumor tissues (Anderton et al., 2017). Moreover, in this type of cancer, miR-22 targets TfR1 and inhibits cell cycle progression and growth (Greene et al., 2013). Besides, miR-152 is another miRNA that regulates ferroptosis in liver cancer cells (Huang et al., 2010). Another experiment in liver cancer cells shows the role of miR-503 in reduction of intracellular levels of SOD and glutathione (Wang et al., 2014).

The role of miRNAs in the regulation of ferroptosis has also been assessed in colorectal cancer cells. In this type of cancer, miR-24-2 levels has been inversely correlated with the levels of superoxide dismutase (SOD) (He et al., 2018). Moreover, induction of ROS by GT-094 has been found to be correlated with modulation of the miR-27a:ZBTB10-Sp1/Sp3/Sp4 axis (Pathi et al., 2011). miR-145 and miR-149 are two other miRNAs that affect expression of TFR1 and DMT1 in colorectal cancer cells (Hamara et al., 2013).

Lung cancer is another type of cancer in which the role of miRNAs in the regulation of ferroptosis has been vastly investigated. For instance, miR-155 silencing has been shown to inhibit GST-π expression in A549/dox cells. miR-155 induces doxorubicin resistance *via* modulation of drug transportation and drug metabolism (Lv et al., 2016). miR-196a is another miRNA that has an indirect effect on ferrptosis. Suppression of this miRNA has suppressed stem cell self-renewal capacity, tumor growth and tumorigenicity through enhancement of expression of GPX3 (Liu et al., 2019). Moreover, miR-302a-3p has been found to induce ferroptosis in lung cancer cells *via* targeting ferroportin (Wei et al., 2021). Besides, miR-324-3p enhances cisplatin-induced ferroptosis in lung cancer cells (Deng et al., 2021).

Therefore, the effects of miRNAs on ferroptosis can be regarded as a mechanism for induction/prevention of different malignancies. Moreover, modulation of expression of ferroptosis-related miRNAs can be regarded as a potential treatment strategy for cancers. Table 1 shows the role of miRNAs in the regulation of ferroptosis in cancers.

**TABLE 1:** miRNAs effect on ferroptosis in cancers.

Associated cancer type	Cell line	Study type	Upstream of miRNA	miRNA	Downstream target of miRNA	Impact on ferroptosis	Study highlights	Reference
Melanoma	A375, G-361	*In vitro*	-	miR-9	GOT1	Inhibitory	miR-9 overexpression suppressed erastin- and RSL3-induced ferroptosis through Gln	Zhang et al. (2018)
Kaposi’s sarcoma	RAW 264.7 cells	Cell culture	-	miR-K12-11	xCT	Indirect inhibitory effect	KSHV miRNAs can increase expression of xCT by macrophage and endothelial cells, mainly *via* miR-K12-11 inhibition of BACH-1, a gene that can promote ferroptosis	Qin et al. (2010)
Prostate cancer	PrEC, PrSC, PZ-HPV-7, HPV-18, LNCaP, DU-145, PC3 (ATCC)	Cell culture, animal models	-	miR-17	GPX2	Indirect effect	miR-17* can inhibit important primary mitochondrial antioxidant enzymes, namely MnSOD, GPX2 and TrxR2	Xu et al. (2010a)
Prostate cancer	PC-3/22Rv1	Cell culture, animal models	-	miR-17-3p	GPX2	Indirect effect	Inhibition of antioxidants by miR-17-3p enhances ROS and radiotherapeutic efficiency in cancer treatment	Xu et al. (2018)
Multiple myeloma	ARP1 and OCI-MY5	Cell culture, Animal models	Nrf2	miR-17-5p	FPN	Indirect effect	Nrf2 enhances FPN1 transcription *via* promoter binding and suppresses miR-17-5p	Kong et al. (2019)
Glioma	U87	*In vitro*/*In vivo*	-	miR-18a	ALOXE3	Inhibitory	miR-18a targets and suppresses ALOXE3 and made glioblastoma cells resistant to p53-induced ferroptosis	Yang et al. (2021b)
Hepatocellular carcinoma	LT2-MYC	Cell culture, animal models	-	miR-18a	GCLC	Indirect effect	miR-18a reduces expression of GCLC- a gene that regulates biosynthesis of glutathione. miR-18a also reduces GSH levels in tumor tissues	Anderton et al. (2017)
Colorectal cancer	-	Clinical samples	-	miR-19a	DMT1	Indirect effect	miR-194 levels have been associated with ferroportin concentrations	Hamara et al. (2013)
Lung cancer	Huh7 and NSCLC	Cell culture	-	miR-20a	FPN	Indirect effect	Downregulation of FPN by miR-20a can result in enhancement of iron pool thus providing additional iron for metabolic process	(Babu and Muckenthaler 2016)
Lung cancer	A549 and A549/DDP	Cell culture	-	miR-21	-	Indirect effect	Expression levels of cystathione and GSH in A549/DDP cells were decreased after miR-21 silencing.	Dong et al. (2015)
Liver cancer	-	Cell culture	-	miR-22	TFR1	Indirect effect	miR-22 targets TfR1, and inhibits cell cycle progression and growth	Greene et al. (2013)
Colorectal cancer	-	Clinical samples	-	miR-24–2	-	Indirect effect	miR-24–2 levels were inversely correlated with the levels of superoxide dismutase (SOD)	He et al. (2018)
Bladder cancer	EJ/T24 and RT112	Cell culture	-	miR-27a	SLC7A11	Indirect effect	Alterations in miR-27a levels are involved in cisplatin resistance in bladder cancer through modulating the expression of the SLC7A11 and intracellular GSH.	Drayton et al. (2014)
Colorectal cancer	RKO and SW480	Cell culture	GT-094	miR-27a	ZBTB10	Indirect effect	Induction of ROS by GT-094 is correlated with modulation of the miR-27a:ZBTB10-Sp1/Sp3/Sp4 axis	Pathi et al. (2011)
Lung cancer	-	Clinical samples	-	miR-29	IREB2	Indirect effect	The miRNA binding site rs1062980 might change IREB2 expression *via* affecting miR-29a binding. This SNP can affect risk of lung cancer	Zhang et al. (2017)
Colorectal cancer	-	Clinical samples	-	miR-31	TFR1	Indirect effect	mRNA levels of TfR1are associated with miR-31 levels	Hamara et al. (2013)
Prostate cancer	LNCaP	Cell culture	-	miR-34b	MYC	Indirect effect	miR-34b and miR-34c can decrease c-Myc protein expression in prostate cells	Benassi et al. (2013)
Prostate cancer	1E8 and 2B4	Cell culture, animal models	-	miR-92b-5p	GST	Indirect effect	miR-92b-5p targets GSTM3, which is involved in the detoxification of electrophilic compounds by conjugation with glutathione	Ma et al. (2019)
Breast cancer	MDA-MB-231, T47D	*In vitro*/*In vivo*	CircRHOT1	miR-106a-5p	STAT3	inhibition	miR-106a-5p induced ferroptosis by targeting STAT3 in breast cancer cells	Zhang et al. (2021a)
Chronic lymphocytic leukemia	ATCC. MEC1 and MEC2	Cell culture	-	miR-125b	-	Indirect effect	miR-125b affects metabolism of glucose, glutathione, lipid, and glycerolipid	Tili et al. (2012)
Prostate cancer	PC3, DU145	*In vitro*/*In vivo*	LncOIP5-AS1	miR-128-3p	SLC7A11	inhibition	OIP5-AS1 inhibited ferroptosis under chronic exposure to Cd through targeting miR-128-3p/SLC7A11 signaling	Zhang et al. (2021b)
Colorectal cancer	HCT116, HT-29	Cell culture	-	miR-129-5P	GST	Indirect effect	miR-129-5p has demonstrated dissimilar pattern of reduction in the resistant SW480 and HCT116 cell lines	Ghanbarian et al. (2018)
Ovarian cancer	A2780	Cell culture	-	miR-130b	GST-π	Indirect effect	miR-130b can decrease recurrence, invasion, and metastasis of ovarian cancer	Zong et al. (2014)
Melanoma	A375, G-361	*In vitro*/*In vivo*	-	miR-130b-3p	DKK1	inhibition	miR-130b 3p suppressed erastin or RSL3 induced ferroptosis	Liao et al. (2021)
Breast cancer	MCF-7	Cell culture	-	miR-133a	FTL	Indirect effect	Decrease in FTL protein levels by miR-133a enhances sensitivity of MCF-7/DOX and MCF-7/CDDP cells to doxorubicin and cisplatin	Chekhun et al. (2013)
Colorectal cancer	-	Clinical samples	-	miR-133a	FTL	Indirect effect	Levels of Fn have been negatively associated with IRP1 transcript levels, while positively correlated with expression of miR-133a	Hamara et al. (2013)
Melanoma	A375, G-361	*In vitro*/*In vivo*	-	miR-137	SLC1A5	inhibition	miR-137 decreased glutamine uptake, MDA accumulation and inhibited erastin-induced ferroptosis	Luo et al. (2018)
Colorectal cancer	-	Clinical samples	-	miR-141	TFR1	Indirect effect	miR-141 can affect expression of TFR1	Hamara et al. (2013)
Prostate cancer	-	Cell culture, animal models	-	miR-144	GST	Indirect effect	miR-144 regulates expression of GSTP1	Singh et al. (2015)
Colorectal cancer	-	Clinical samples	-	miR-145	TFR1	Indirect effect	miR-145 can affect expression of TFR1	Hamara et al. (2013)
Colorectal cancer	-	Clinical samples	-	miR-149	DMT1	Indirect effect	miR-149 can affect expression of DMT1	Hamara et al. (2013)
Hepatocellular carcinoma	HepG2, HepG2.2.15, Huh-7, LO2, and Hepa1-6	Cell culture	-	miR-152	DNA methyltransferase 1	Indirect effect	Inhibition of miR-152 could enhance GSTP1 expression	Huang et al. (2010)
Hepatocellular carcinoma	SK-HEP1, PLC/PRF/5, Hep3B, and HepG2	Cell culture, animal models	-	miR-152	TFRC	Indirect effect	Up-regulation of TFRC can be involved in cancer-related abnormalities in cellular iron metabolism during liver carcinogenesis. Over-expression of TFRC can be due to down-regulation of miR-152	Kindrat et al. (2016)
Prostate cancer	-	Cell culture, animal models	-	miR-153–1/2	GSTP1	Indirect effect	miR-153–1/2 regulate expression of GSTP1	Singh et al. (2015)
Lung cancer	A549	Cell culture	-	miR-155	GST-π	Indirect effect	miR-155 silencing inhibited GST-π expression in A549/dox cells. miR-155 induces doxorubicin resistance *via* modulation of drug transportation and drug metabolism	Lv et al. (2016)
Colorectal cancer	-	Clinical samples	-	miR-182	TFR1	Indirect effect	miR-182 can affect expression of TFR1	Hamara et al. (2013)
Ovarian cancer	OVCAR3, A2780, A2780/DDP, and A2780/Taxol	Cell culture	-	miR-186	GST	Indirect effect	miR-186 has a role in induction sensitivity to paclitaxel and cisplatin through regulation of expression of ABCB1	Sun et al. (2015)
Colorectal cancer	-	Clinical samples	-	miR-194	FPN1	Indirect effect	miR-194 can affect expression of FPN1 ferroportin 1	Hamara et al. (2013)
NSCLC	A549, H460, H1975, H1650, HCC827	Cell culture, animal models	-	miR-196a	GPX3	Indirect effect	miR-196a suppression suppressed NSCLC stem cell self-renewal capacity, stemness, tumor growth and tumorigenicity through enhancement of expression of GPX3.	Liu et al. (2019)
Renal cancer	RCC4 and 786-O	Cell culture	-	miR-210	ISCU	Indirect effect	miR-210 level is inversely correlated with ISCU levels	McCormick et al. (2013)
oropharyngeal squamous cell carcinomas	SCC2 and SCC38	Cell culture	-	miR-210	ISCU	Indirect effect	miR-210 targets ISCU.	Sáenz-de-Santa-María et al. (2017)
Hepatoma	HepG2, Hep3B, Hep3B rat	*In vitro*/*In vivo*	-	miR-214-3p	ATF4	enhancement	miR-214 enhanced erastin-induced ferroptosis by targeting ATF4	Bai et al. (2020)
Bladder cancer	T24 and EJ	Cell culture	-	miR-218	GCL	Indirect effect	Over-expression of miR-218 significantly reduced the rate of glucose uptake and total level of GSH and enhanced the chemo-sensitivity of bladder cancer to cisplatin	Al-Kafaji et al. (2017)
NSCLC	A549, H358	-	-	miR-302a-3p	FPN	promote	miR-302a-3p induced ferroptosis *via* targeting ferroportin	Wei et al. (2021)
Colorectal cancer	HCT116, HT-29	Cell culture	-	miR-302c-5p	GST	Indirect effect	Over-expression of ABCB1 by miR-302c-5p defines poor response to oxaliplatin	Ghanbarian et al. (2018)
NSCLC	A549, DDP	*In vitro*	-	miR-324-3p	GPX4	enhancement	miR-324-3p enhanced cisplatin-induced ferroptosis	(Deng et al., 2021)
Glioma	U87MG, U251MG, T98G, U373MG, and A172	Cell culture	-	miR-326	PKM2	Indirect effect	PKM2 silencing diminished ATP and glutathione levels and activated AMPK. PKM2 is a target of the tumor-suppressive miR-326 and a potential therapeutic target in gliomas	Kefas et al. (2010)
Rectal cancer	-	-	CircABCB10	miR-326	CCL5	inhibition	miR-326inhibits ferroptosis and apoptosis through regulation of CCL5	Xian et al. (2020)
NSCLC	A549, H1299	*In vitro*/*In vivo*	lncMT1DP	miR-365a-3p	NRF2	enhancement	miR-365a-3p was enhanced and NRF2 was suppressed in MT1DP-overexpressing A549 and H1299 cells	Gai et al. (2020)
Gastric cancer	SGC-7901, BGC-823	*In vitro*/*In vivo*	-	miR-375	SLC7A11	enhancement	miR-375 triggered SLC7A11-dependent ferroptosis	Ni et al. (2021a)
Cervical cancer	CaSki, HeLa, HcerEpic	*In vitro*/*In vivo*	circRNA_000479	miR-375, miR-409-3P, miR-515-5p	SLC7A11	inhibition	circEPSTI1-miR-375/409-3P/515-5p-SLC7A11 axis promoted the cervical cancer cell proliferation and its knockdown induced ferroptosis	Wu et al. (2021)
Ovarian cancer	-	-	-	miR-424-5p	ACSL4	inhibition	miR-424-5p inhibits ferroptosis *via* targeting ACSL4	Ma et al. (2020)
Glioma	U87	Cell culture, animal models	-	miR-449a	CISD2	Indirect effect	miR-449a targets CISD2 3′-UTR in U87 cells	Sun et al. (2017)
Prostate cancer	PC3 and DU145	Cell culture, animal models	-	miR-492	FPN	Indirect effect	miR-492 is involved in modulating MZF-1-mediated regulation on FPN and growth of prostate cancer cells	Chen et al. (2015)
Cervical cancer	HeLa, HeLa/DDP, and SiHa	Cell culture	-	miR-497	-	Indirect effect	miR-497/TKT axis affects GSH and ROS levels, and enhance DDP chemoresistance in cervical cancer	Yang et al. (2016)
Hemangioma	HemECs	*In vitro*	lncMEG8	miR-497-5p	NOTCH2	inhibition	MEG8 silencing has suppressed proliferation and induced the ferroptosis by regulating miR-497-5p/NOTCH2 axis	Ma et al. (2021)
Hepatocellular carcinoma	HepG2	Cell culture	-	miR-503	-	Indirect effect	miR-503 increases apoptosis, blocks the cell cycle transition and reduces intracellular levels of SOD and glutathione	Wang et al. (2014)
Colorectal cancer and lung cancer	A549/CDDP and SPC-A-1	Cell culture	-	miR-513a-3p	GST	Indirect effect	miR-513a-3p can sensitize human lung cancer cells to cisplatin by targeting GSTP1	Zhang et al. (2012)
Oral squamous cell carcinoma	CAL27, SCC15	*In vitro*/*In vivo*	circFNDC3B	miR-520days-5p	SLC7A11	inhibition	circFNDC3B attenuated ferroptosis by regulating miR-520days-5p/SLC7A11 axis	Yang et al. (2021a)
Gastric cancer	SGC7901, MGC803, MKN45	*In vitro*/*In vivo*	USP7, hnRNPA1	miR-522	ALOX15	inhibition	miR-522 decreased ALOX15 and lipid-ROS and inhibited ferroptosis	Zhang et al. (2020a)
Hepatocellular carcinoma	THLE-2, HuH-7, HCCLM3	*In vitro*/*In vivo*	CircIL4R	miR-541-3p	GPX4	inhibition	Down-regulation of miR-541-3p relieved the ferroptosis promotion caused by circIL4R knockdown	Xu et al. (2020)
Thyroid cancer	Nthy-ori 3–1, FTC133, TPC-1	*In vitro*/*In vivo*	Circ_0067934	miR-545-3p	SLC7A11	inhibition	miR-545-3p induced ferroptosis by targeting SLC7A11	Wang et al. (2021a)
Prostate cancer	-	Cell culture, animal models	-	miR-590-3p/5p	GST	Indirect effect	miR-590-3p/5p regulate expression of GSTP1	Singh et al. (2015)
Prostate cancer	DU145 and PC3	Cell culture, animal models	-	miR-638	FTH-1	Indirect effect	miR-638 overexpression reduced FTH1 protein expression	Chan et al. (2018)
Glioblastoma	U87MG, A172	-	-	miR-670-3p	ACSL4	inhibition	miR-670 inhibits ferroptosis *via* influencing expression of ACSL4	Bao et al. (2021)
Colorectal cancer	-	Clinical samples	-	miR-758	TFR1	Indirect effect	miR-758 can affect expression of TFR1 transferrin receptor 1	Hamara et al. (2013)
Glioma	LN229, U251, NHA	*In vitro*/*In vivo*	circ-TTBK2	miR-761	ITGB8	inhibition	circ-TTBK2 knockdown or miR-761 increase could promote ferroptosis	Zhang et al. (2020c)
Colorectal cancer	HCT116, SW620, SW480	*In vitro*/*In vivo*	Circ_0007142	miR-874-3p	GDPD5	inhibition	circ_0007142 expression inhibition motivated CRC ferroptosis. Overexpression of miR-874-3p promoted ferroptosis	Wang et al. (2021b)
Hepatocellular carcinoma	SMMC-7721, QGY-7703	*In vitro*/*In vivo*	circ_0013731	miR-877-3p	SLC7A11	inhibition	circ_0013731 mediated by E2F1 suppressed the ferroptosis *via* miR-877-3p/SLC7A11 axis	Fang et al. (2021)
Lung cancer	A549, H1975, H1650	Cell culture	-	miR-921	GPX3	Indirect effect	miR-921 suppresses GPx3 expression in lung cancer cells	Choi et al. (2019)
Papillary thyroid cancer	KAT-5, TPC-1	*In vitro*/*In vivo*	circKIF4A	miR-1231	GPX4	inhibition	GPX4 was the target of miR-1231. circKIF4A could enhance expression of GPX4.	Chen et al. (2021b)
Medulloblastoma	-	-	-	miR-1253	ABCB7	enhancement	Overexpression of miR-1253 resulted in downregulation of ABCB7 and GPX4	Kanchan et al. (2021)
Hepatocellular carcinoma	LO2, HepG2, BEL-7402, MHCC-97H	*In vitro*/*In vivo*	Circ0097009	miR-1261	SLC7A11	inhibition	Circ0097009 silencing enhances ferroptosis *via* the circ0097009/miR-1261/SLC7A11 axis	Lyu et al. (2021)
Colorectal cancer	HCT116, HT-29	Cell culture	-	miR-3664-5p	GST	Indirect effect	GSTP1 levels have been correlated directly with miR-3664-5p	Ghanbarian et al. (2018)
NSCLC	A549	*In vitro*/*In vivo*	-	miR-4443	METTL3	inhibition	Overexpression of miR-4443 inhibited cisplatin-induced ferroptosis	(Song et al., 2021c)
GI cancer	OE33, MKN45, STKM2	*In vitro*	-	miR-4715-3p	AURKA	enhancement	miR-4715-3p induced ferroptosis by reducing GPX4 in a AURKA-dependent mechanism	Gomaa et al. (2019)

**FIGURE 1:**
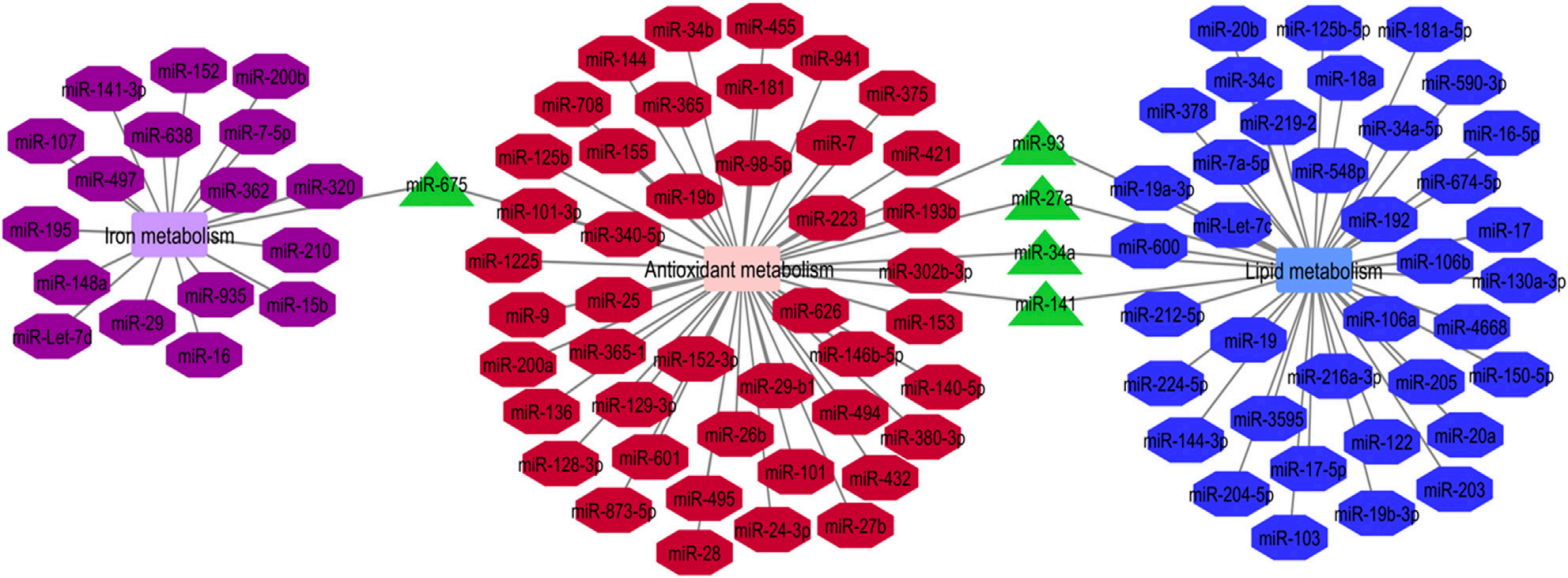
Iron metabolism, antioxidant metabolism and lipid metabolism are the pivotal mechanisms in the ferroptosis process. The mentioned metabolisms-related miRNAs network was represented by Cytoscape software. The common miRNAs (miR-675, miR-93, miR-27a, miR-34a and miR-141) in these three networks with the most degree and betweenness centrality as the key miRNAs are shown by green triangles (Luo et al., 2021a).

Cumulatively, miRNAs participating in the regulation of iron metabolism, antioxidant metabolism and lipid metabolism are associated with ferroptosis process (Luo et al., 2021a). We have constructed the network between these miRNAs using the Cytoscape software. Five miRNAs, namely miR-675, miR-93, miR-27a, miR-34a and miR-141 have been found to be involved in these three metabolic pathways (Figure 1). Pre-miR-675 is produced by lncRNA H19. FTH1 silencing upregulates expressions of H19 and its cognate miR-675. Activation of H19/miR-675 participates in the FTH1 silencing-related alterations in iron metabolism (Di Sanzo et al., 2018). miR-93 regulates expression of NRF2 and has a role in breast carcinogenesis (Singh et al., 2013). miR-27a directly inhibits expression of SCD1 (Drayton et al., 2014). miR-34a directly suppresses expression of ACSL4 (Jiang et al., 2020). Finally, miR-141 inhibits Nrf2 signaling through targeting Keap1 (Wu et al., 2018).

### miRNAs effects on ferroptosis in non-malignant conditions

**FIGURE 2:**
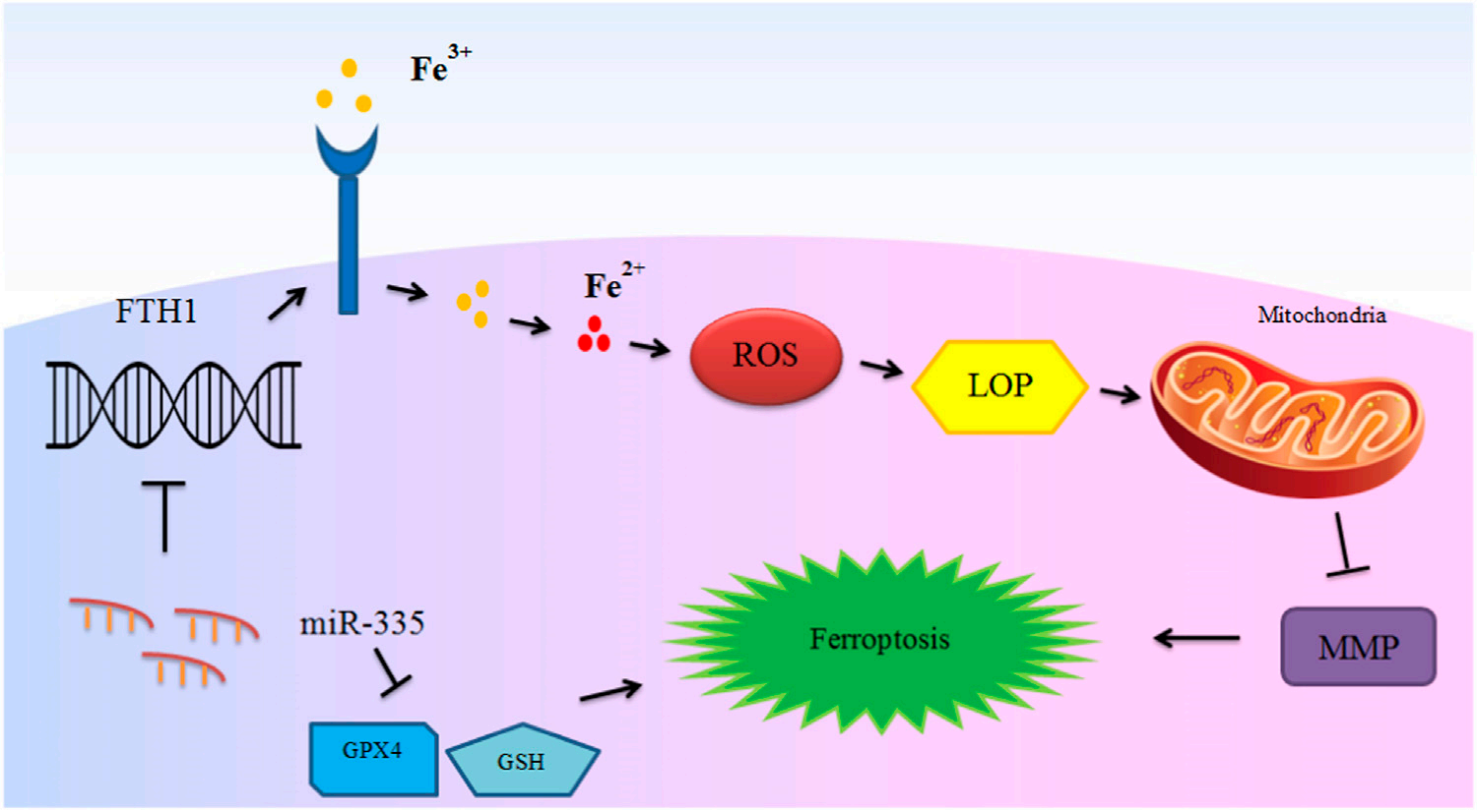
The mechanism of ferroptosis induction in Parkinson’s disease by miR-335. miR-335 targets FTH1 and degrades it to promote iron release, lipid peroxidation and reactive oxygen species (ROS) accumulation, and decreases mitochondrial membrane potential (MMP) and intensify ferroptosis and PD pathology. Although glutathione peroxidase 4 (GPX4) is not directly targeted by miR-335, up-regulation of miR-335 also leads to reduction of the levels of this ferroptosis marker protein (Li X. et al., 2021).

miRNAs have important roles in the ferroptosis in non-malignant conditions. Parkinson’s disease is an example of disorders in which the role of miRNAs in the regulation of ferroptosis has been assessed. An experiment in this field has shown down-regulation of GPX4 in the animal model of this disorder in association with down-regulation of FTH1 and over-expression of miR-335. miR-335 mimic could decrease expression of FTH1, increase ferroptosis and facilitate progression of Parkinson’s disease. Mechanistically, miR-335 targets 3′-UTR of FTH1. FTH1 silencing in 6-OHDA-induced cells has increased the pro-ferroptosis impact of miR-335 and promoted pathologic events in the course of Parkinson’s disease. In fact, miR-335 enhances ferroptosis *via* reduction of FTH1 and subsequent enhancement of iron release, lipid peroxidation and ROS buildup, while decreasing mitochondrial membrane potential (Li X. et al., 2021). Figure 2 depicts this process.

Another experiment has shown aberrant expression of IL-6 and its receptor in cartilage samples of patients with intervertebral disc degeneration. Notably, IL-6 could down-regulate expression of miR-10a-5p, leading to derepression of IL-6R expression. IL-6 has a role in induction of ferroptosis in cartilage cells through stimulating oxidative stress and upsetting iron homeostasis. Up-regulation of miR-10a-5p could decrease IL-6R levels and attenuate IL-6-associated ferroptosis to some extent (Bin et al., 2021).

Expression of miR-15a-5p has been shown to be increased in acute myocardial infarction. miR-15a-5p silencing has decreased mortality of myocardial cells in hypoxic conditions. Notably, GPX4 has been identified as the direct target of miR-15a-5p. Up-regulation of miR-15a-5p has enhanced ferroptosis and intensified myocardial cell damage during hypoxia. Knock-down of the transcription factor Egr-1 has led to down-regulation of miR-15a-5p, and subsequent up-regulation of GPX4, which results in reduction of ferroptosis and alleviation of myocardial damage (Fan et al., 2021).

miR-17-92 is another miRNA which modulates ferroptosis. This miRNA has been shown to protect endothelial cells from erastin-associated ferroptosis. In fact, over-expression of miR-17-92 can reduce erastin-associated growth suppression and ROS production in endothelial cells. Mechanistically, miR-17-92 exerts its effects through suppression of Zinc lipoprotein A20 expression. miR-17-92 up-regulation or A20 suppression has enhanced expression of ACSL4 endothelial cells (Xiao F. J. et al., 2019).

Ferroptosis in animal model of intracerebral hemorrhage has been associated with reduced levels of miR-19b-3p and enhancement of IRP2 levels. Expression of IRP2 as a direct target of miR-19b-3p has been suppressed by miR-19b-3p mimic-transfected adipose-derived stem cells. These exosomes could also attenuate hemin-associated cell damage and ferroptosis, thus improving neurologic function in the effected animals (Yi and Tang 2021). Taken together, ferroptosis-associated miRNAs are involved in the pathogenesis of a variety of non-malignant conditions, such as intervertebral disc degeneration, acute myocardial infarction, vascular diseases, intracerebral hemorrhage and preeclampsia. Table 2 shows the role of miRNAs in regulation of ferroptosis in non-malignant conditions.

**TABLE 2:** miRNAs effect on ferroptosis in non-malignant conditions.

Condition	Cell line	Study type	Upstream of miRNA	miRNA	Downstream of miRNA	Effect of on ferroptosis	Study highlights	Reference
-	HeLa, SAS	*In vitro*	-	miR-7-5p	ALOX12	inhibition	Knockdown of miR-7-5p leads to enhancement of the ferroptosis marker ALOX12 gene expression	Tomita et al. (2021)
Intervertebral disc degeneration	-	*In vitro*	IL-6	miR-10a-5p	IL-6R	enhancement	IL-6R derepressing from miR-10a-5p enhanced IL-6 signaling	Bin et al. (2021)
Acute myocardial infarction	-	-	Egr-1	miR-15a-5p	GPX4	enhancement	miR-15a-5p enhances ferroptosis through regulation of GPX4	Fan et al. (2021)
Vascular disease	HUVEC	*In vitro*	-	miR-17–92	A20	inhibition	miR-17–92 protected the HUVEC cells from erastin-induced ferroptosis maybe through miR-17–92/A20/ACSL4 axis	Xiao et al. (2019)
Intracerebral hemorrhage	adipose-derived stem cells(ADSCs)	*In vitro*/*In vivo*	-	Exo-miR-19b-3p	IRP2	inhibition	Exosomal miR-19b-3p originated from ADSCs could abrogate Hemin-associated ferroptosis	(Yi and Tang 2021)
Acute myocardial infarction	HUCB-MSCs	*In vitro*/*In vivo*	-	Exo-miR-23a-3p	DMT1	inhibition	HUCB-MSCs-derived miR-23a-3p-expressing exosomes suppress ferroptosis	Song et al. (2021b)
Preeclampsia	HTR-8/SVneo, TEV-1	*In vitro*/*In vivo*	-	miR-30-5p	Pax3 and SLC7A11	enhancement	Upregulation of miR-30b-5p downregulated SLC7A11, Pax3, and Pax3-downstream target, FPN1, and induces ferroptosis	(Zhang et al., 2020b)
Myocardial infarction	-	-	-	miR-30d	ATG5	enhancement	miR-30days suppresses cardiomyocytes autophagy and promote ferroptosis	Tang et al. (2020)
-	A549, L78, NCI–H460, GLC-82, SPC-A1, PC9, BEAS-2B	*In vitro*/*In vivo*	-	miR-101-3p	TBLR1	enhancement	Activation of the miR-101–3p/TBLR1 axis directly recovered tumor cell ferroptosis	Luo et al. (2021b)
Intracerebral hemorrhage	BMVECs	*In vitro*/*In vivo*	lncH19	miR-106b-5p	ACSL4	enhancement	H19 silencing enhances cell proliferation and suppresses BMVECs ferroptosis	Chen et al. (2021a)
Intracerebral hemorrhage	-	*In vitro*/*In vivo*	-	miR-124	FPN	inhibition	miR-124/Fpn signaling mediates neuron death post-ICH through apoptosis and ferroptosis	Bao et al. (2020)
Myocardial I/R injury	H9C2	*In vitro*/*In vivo*	-	miR-135b-3p	GPX4	promote	miR-135b-3p promoted cellular ferroptosis by downregulating GPX4 expression	Sun et al. (2021b)
Hemorrhagic Stroke	SH-SY5Y	*In vitro*	-	Exo-miR-137	COX2/PGE2	inhibition	EXsmiR-137 suppresses oxyHb-induced ferroptosis	Li et al. (2020)
Atrial fibrillation	-	*In vitro*	-	miR-143-3p	GOT1	inhibition	Overexpression of miR-143-3p inhibited ferroptosis	Song et al. (2021)
Pulmonary fibrosis	HFL1	*In vitro*/*In vivo*	lncZFAS1	miR-150-5p	SLC38A1	enhancement	Overexpression of lncRNA ZFAS1 increased ferroptosis through decreasing the inhibitory effect of miR-150-5p on SLC38A1 expression	Ni et al. (2021)
MI	H9c2, HEK-293 T	*In vitro*	-	miR-190a-5p	GLS2	inhibition	Upregulation of miR-190a-5p inhibited ferroptosis induced by erastin and RSL3	Zhou et al. (2021)
Atherosclerosis	Endothelial progenitor cells	*In vitro*/*In vivo*	-	EV-miR-199a-3p	SP1	inhibition	EPC-EVs carrying miR-199a-3p have an impact on the function of ECs and inhibited ferroptosis of ECs by targeting SP1	(Li et al., 2021a)
TBI	HT-22, Neuro-2a	*In vitro*/*In vivo*	-	miR-212-5p	Ptgs2	inhibition	miR-212-5p suppressed the ferroptotic neuronal death partly by targeting Ptgs2	Xiao et al. (2019b)
Brain ischemia/reperfusion	PC12 rat	*In vitro*/*In vivo*	lncPVT1	miR-214	TP53, TFR1, PVT1	enhancement	PVT1 silencing or miR-214 up-regulation could decrease p53 levels and increase SLC7A11 levels	Lu et al. (2020)
Heart Failure	HL-1	*In vitro*	circSnx12	miR-224-5p	FTH1	inhibit	Low circSnx12 expression and high miR-224-5p expression induced ferroptosis	Zheng et al. (2021)
PD	PC12	*In vitro*/*In vivo*	-	miR-335	FTH1	enhancement	miR-335 promoted ferroptosis in PD by inhibiting FTH1 expression	Moradi et al. (2021)
Chronic heart failure	HL-1	*In vitro*/*In vivo*	-	miR-351	MLK3	inhibition	Enhancement of expression of miR-351 improved cardiac function in animal models	Wang et al. (2020)
I/R-induced renal injury	HK-2, TCMK-1 rat	*In vitro*/*In vivo*	-	miR-182-5p	GPX4	enhancement	miR-182-5p and miR-378a-3p promoted ferroptosis in the renal epithelial cells by suppressing GPX4 and SLC7A11, respectively	Ding et al. (2020)
				miR-378a-3p	SLC7A11			

### Effects of different treatments on expression of ferroptosis-associated miRNAs

A number of drugs and treatments have been found to influence course of disorders through affecting expression of ferroptosis-associated miRNAs. For instance, experiments in animal models of intracerebral hemorrhage have shown that acupuncture can amend neuron cells death, inflammatory responses, and ferroptosis through downregulation of miR-23a-3p. The effects of acupuncture on alleviation of ferroptosis and reduction of miR-23a-3p expression have been verified by the observed enhancemnet of nuclear translocation of NFE2L2 and expression of heme oxygenase-1 and glutathione peroxidase 4 as well as reduction of iron and malondialdehyde levels and decrease in the accumulation of reactive oxygen species. Furthermore, antagomiR-23a-3p could inhibit the intracerebral hemorrhage-induced enhancemnet of Fluoro-Jade B-positive cells, production of proinflammatory cytokines, ferroptosis, and activity of NFE2L2. Mechanistically, miR-23a-3p has binding site on NFE2L2 (Kong et al., 2021). On the other hand, isorhynchophylline has been shown to ameliorate ferroptosis-associated nerve injury in the context of intracerebral hemorrhage through modulation of miR-122-5p (Zhao et al., 2021). Experiments in mouse hippocampal HT-22 cells exposed to ferric ammonium citrate alone or together with Isorhynchophylline have shown that Isorhynchophylline reduces the ferric ammonium citrate-associated cell injury. Isorhynchophylline also reduces the ferric ammonium citrate-induced reduction of miR-122-5p expression and ameliorates ferroptosis. Besides, miR-122-5p inhibitor could diminish the protective effect of Isorhynchophylline against ammonium citrate-associated ferroptosis in these cells. Mechanistically, miR-122-5p targets TP53, and restoration of TP53 attenuates the effect of miR-122-5p on ferroptotic markers and expression of SLC7A11. Taken together, miR-122-5p/TP53/SLC7A11 axis has been suggested as a potential mechanism in the etiology of intracranial hemorrhage (Zhao et al., 2021).

Moreover, metformin can induce ferroptosis of breast cancer cells through influencing expression of the GPX4 targeting miRNA miR-324-3p. Up-regulation of miR-324-3p has suppressed viability of breast cancer cells. In fact, metformin can be regarded as a potential anti-cancer agent *via* activation of ferroptosis (Hou et al., 2021). Similarly, lidocaine and levobupivacaine enhance ferroptosis of cancer cells through targeting miR-382-5p (Sun D. et al., 2021) and miR-489-3p (Mao et al., 2021), respectively.

Table 3 shows the effects of different treatments on expression of ferroptosis-associated miRNAs.

**TABLE 3:** Effects of drugs on ferroptosis-associated miRNAs.

Disease	Cell line	Study type	Drug/Treatment	Regulation of miRNA by treatment	miRNA	Target	Effect of drug on ferroptosis	Study highlights	Reference
Intracerebral hemorrhage	-	*In vivo*	Acupuncture	↓	miR-23a-3p	NFE2L2	inhibition	Baihui-penetrating-Qubin acupuncture treatment inhibited ferroptosis after ICH *via* down-regulating miR-23a-3p	Kong et al. (2021)
Intracerebral hemorrhage	HT-22	*In vitro*/*In vivo*	Isorhynchophylline	↑	miR-122-5p	TP53	inhibition	miR-122-5p Suppresses FAC-Induced Ferroptosis by Targeting TP53	Zhao et al. (2021)
Breast cancer	MDA-MB-231, MCF-7	*In vitro*/*In vivo*	Metformin	↑	miR-324-3p	GPX4	enhancement	miR-324-3p induced ferroptosis *via* directly targeting GPX4	Hou et al. (2021)
Ovarian and Breast cancer	SKOV-3, T47D	*In vitro*/*In vivo*	Lidocaine	↑	miR-382-5p	SLC7A11	enhancement	Lidocaine Inhibited SLC7A11 Expression by Upregulating miR-382-5p	Sun et al. (2021a)
Gastric cancer	GES-1, HGC27, SGC7901	*In vitro*/*In vivo*	Levobupivacaine	↑	miR-489-3p	SLC7A11	enhancement	Levobupivacaine induces ferroptosis of gastric cancer cells through miR-489-3p/SLC7A11 axis	Mao et al. (2021)

## Discussion

Several miRNAs have been found to affect ferroptosis through binding with 3′-UTR of genes participating in this process. GOT1, GPX2, GPX3, GPX4, FPN, GSH, GST, FTL, TFR1 and NRF2 are examples of ferroptosis-associated molecules which are regulated by miRNAs. Moreover, a number of miRNAs can affect ferroptosis through indirect routes. For instance, miR-152 can reduce expression of DNA methyltransferase one leading to global DNA hypomethylation and enhancement of expression of GSTP1 (Huang et al., 2010).

Since ferroptosis can eradicate cancer cells in an independent way from apoptosis (Zhang X. et al., 2020), identification of the role of miRNAs in this process can propose new ways for combatting cancer progression. It is worth mentioning that some of above-mentioned miRNAs that regulate ferroptosis, have additional roles in the regulation of other types of cell death. Thus, these miRNAs can induce cancer cell death from different routes.

Bioinformatics tools have facilitated identification of miRNAs with highest involvement in the ferroptosis, thus proposing the most appropriate targets for management of ferroptosis-associated disorders.

A number of long non-coding RNAs and circRNAs have been found to affect expression of ferroptosis-related miRNAs. CircRHOT1, circABCB10, circRNA_000479, circFNDC3B, circIL4R, circ_0067934, circ-TTBK2, circ_0007142, circ_0013731, circKIF4A, circ0097009, lncOIP5-AS1, lncMT1DP and lncMEG8 have been recognized as competing endogenous RNAs for miRNAs that partake in the ferroptosis. Therefore, ferroptosis can be regulated by several members of non-coding RNAs.

Notably, acupuncture and a number of drugs such as physcion 8-O-β-glucopyranoside, isorhynchophylline, metformin, lidocaine and levobupivacaine have been shown to affect ferroptosis through modulation of miRNAs. Thus, identification of the role of miRNAs in the regulation of ferroptosis can facilitate design of novel therapeutic agents for treatment of diverse neoplastic or neurodegenerative disorders.

Based on the vast impact of ferroptosis on development of disorders, therapies targeting this process can be proposed as treatment modalities for several disorders including neoplastic and neurodegenerative disorders. Manipulation of expression of ferroptosis-associated miRNAs through different methods is regarded as a potential strategy to affect ferroptosis and intervene with the pathoetiology of mentioned disorders. Since ferroptosis might have opposite effects on the physiology of organs, context-based strategies are needed in this regard. Other issues that should be addressed before incorporation of miRNA-based therapies in the clinical settings are identification of safe and efficient methods for delivery of these kinds of therapies into the specific cells and monitoring the cellular response to these modalities.

Ferroptosis-related miRNAs can alter response of cancer cells to chemotherapeutic modalities. Therefore, manipulation of expression of these miRNAs not only affects the progression and evolution of cancer, but also influences the response to a variety of treatment options. In spite of extensive research on effectiveness of these modalities in cancer cell lines and animal models, there is no clinical trial for appraisal of these methods in the clinical settings. However, it is expected that combination of miRNA-based therapies with conventional or targeted anti-cancer therapies enhances the effectiveness of these therapies. Since cancer cells are heterogeneous in terms of miRNAs signature, it is necessary to have a miRNA profile for each patient before implementation of these novel methods in the clinical settings.

## Abbreviations

reactive oxygen species; (ROS), glutathione peroxidase 4; (GPX4), Kaposi’s sarcoma herpes virus; (KSHV), manganese superoxide dismutase; (MnSOD), thioredoxin reductase-2; (TrxR2), ferroportin; (FPN1), glutamic-oxaloacetic transaminase 1; (GOT1), Glutathione; (GSH), glutathione S-transferase; (GST), Fms-like tyrosine kinase 3; (FTL3), transferrin receptor 1; (TFR1), Nuclear factor erythroid 2-related factor 2; (NRF2).
